# Physical Exercises in the Treatment of Nervous Diseases

**Published:** 1915-01

**Authors:** Theodore Toepel

**Affiliations:** 929-930 Candler Bldg., Atlanta, Ga.


### PHYSICAL EXERCISES IN THE TREATMENT OF NERVOUS DISEASES.

By Theodore Toepel, M. D.

In presenting this paper there is neither time nor necessity for any lengthened account of nervous diseases, I therefore confine myself to those nervous diseases that naturally seek the advice and guidance of the orthopedic surgeon.

Of the various nervous affections amenable to treatment by physical exercise, the functional are those in which the most striking results are obtainable. Since, however, the publication of the classic work by Frenkel, of Switzerland, the treatment of some organic diseases, notably tabetic ataxia, by systematic and graduated exercises, has shown brilliant results, thus bringing much light and encouragement where previously there had been only darkness and despair. Of the organic diseases subject to treatment there are none which afford more satisfactory results than those of locomotor ataxia, and of infantile spinal paralysis.

In the treatment of any of the nervous diseases by exercise, emphasis must be laid upon one meaning of the term in one class of diseases and more emphasis upon another signification of the word in other cases. In the treatment of tabetic ataxia the process is essentially one of re-education; in the treatment of infantile paralysis it is, in a more marked degree, one of development; in the treatment of neurotic disorders, emphasis should be laid upon the word discipline.

### TABETIC ATAXIA.

Briefly, it should be stated here that tabetic ataxia is a well recognized, organic disease of the spinal cord, characterized by peculiar disturbance of gait and difficulty in co-ordinating voluntary movements. The lesion is located in the posterior columns of the cord so that sensation is greatly disturbed or lost. The task which Frenkel sets himself is to make up for this loss by a direct control of the motor functions by attention and re-education.

In order to learn a movement it is essential that the motor stimulus be repeated again and again and that the patient's attention be directed to it. Beginning with the simplest modes of action one proceeds gradually to those more complicated, calling into play the powers of muscular co-ordination until locomotion may become well restored. The treatment of tabetic ataxia is based chiefly upon education of the central nervous system. The unfailing certainty of the improvement of a symptom which has been caused by an organic lesion attaches special interest to the exercise treatment. The symptom to which attention and treatment are directed is a motor disturbance which has its origin not in diminution of the motor power of the muscles, but in a loss of sensation. The result for betterment is brought about by calling into action the cardinal quality of nervous matter to increase in function by exercise, provided that the motor apparatus is intact. The principle employed is identical with that called into play by a healthy person who would learn a complicated combination of difficult movements as in rope dancing.

### INFANTILE SPINAL PARALYSIS

The importance of the treatment of this affection by means of physical exercise has been greatly emphasized because of the fact that there have been several extensive epidemics of the disease in recent years, so that the management and care of these patients has become a very important means in orthopedic practice. This also is an organic affection, one in which the anterior columns of the cord are invaded. The disturbance in tabetic ataxia, as was pointed out, arises from disease of the posterior columns and therefore interferes with sensation. In this disease the disturbance causes a direct interference with the power of locomotion—it is a motor paralysis. Fortunately so far as the paretic element is concerned the tendency is toward recovery or improvement. By the attention that can be given in training these patients very marked improvement can be produced along two or three lines. First, the exercise of disabled groups of muscles tends to prevent their degeneration and aids in a partial restoration of power. Then again other muscles can be used more or less in a substitutional way to take the place and perform the duties of those that were disabled. Finally, education of the higher nerve centers is an important factor. For such treatment the most important variety of the various disabilities which result from infantile paralysis is that in which a complete paraplegia results—where both the lower extremities are completely disabled. The proportion of cases so seriously affected is not large.

### CHOREA.

Chorea is another affection which is distinguished by very great inco-ordination of movement.

In simple chorea, when the child retains a certain measure of control over his movements, simple and rhythmical floor exercises done to command and serve as a sort of discipline for the nerve centers to which little by little the members yield obedience, and the will gradually resumes its control over the muscles.

In more serious cases the disorder of movement is complete and the child is powerless to control even in the slightest degree the agitation of the limbs. In such cases treatment for four or five days is limited to general massage of all the muscles; then passive movements are undertaken.

The limbs are held quiet for a few minutes and then passive movements are given methodically and rhythmically. At the commencement the will of the patient takes no part or even sets up opposition. Then little by little it is felt that the exercised muscles form the habit of association through the efforts readily induced by the operator. At first the will has but a feeble control over the muscular system, but little by little it seems to resume its function and the frequency and disorder of the movements diminish in intensity.

I have observed that in chorea that has become chronic a much longer period of time is required in order to give the patient control over her own movements. In cases of chorea of shorter duration I have seen marked improvement within a week and complete return to self-control within six weeks after the commencement of treatment. Suggestion and example should be employed as consistently as possible. Of course, patients who are having an elevated temperature, or who have acute heart lesions should be excluded from all strenuous exercises. Quite other treatment is then called for.

NEUROTIC AFFECTIONS.

In the ordinary neurotic affections which present themselves, emphasis must be laid upon the disciplinary element of physical exercise. This is best attained by having the patients work together in classes. Patients whose movements both physical and mental have been sluggish, undirected and without positive aim or purpose soon show, under the specific commands of an instructor, that the habits of tardy and purposeless activity can be changed into modes of action which imply a purposeful self-control, quickness and accuracy of response and a keen interest in the attainment of success. Much tact on the part of the teacher is essential to success. Conviction as the work goes on from day to day must become established in the mind of the patient that there is a genuine interest in her case, a desire to help, and entire confidence that success awaits her efforts.

Among the chief causative agents in these neuroses I would name:

A soil suitable for the development of this state, a mentality that has been pre-determined by an unfavorable heredity.

Home life, especially in childhood, the most important factor. Where there is a lack of self-control manifested in dealing with the child and by the parents in their conduct toward each other; where there is a failure to exercise firm, wise, unselfish discipline where untruthfulness is permitted or encouraged; where deceit is practiced toward the children or other members of the family; where in general, the moral regimen of the home has been below par, the seed is being constantly sown, the harvest of which is to be reaped in nerve storms in after years.

The environment even after the period of childhood is passed, though a factor of less importance is one of considerable moment. It is comparatively rare, however, to find that one who has had good upbringing yields to any unfriendly environments in after life.

The lack of self-control manifested in these neurotics makes it our prime duty to employ means and to adduce methods by which we shall enable them to regain self-mastery.

In my judgment it should never be lost sight of that these patients must be taught, whether by suggestion or direct instruction, that they have a responsibility toward their fellows which no other than themselves can sustain. They must eschew selfishness and practice altruism; must be able to look outward and upward rather than encourage self-morbidity by introspection; must execute the instructions of their will and judgment instead of allowing the emotions to hold sway; must realize that habits grow with exercise and their future can only be surrounded by clouds and darkness unless they turn toward the sunshine.

929-930 Candler Bldg., Atlanta, Ga.

